# Eye tracking demonstrates the influence of autistic traits on social attention in a community sample from India

**DOI:** 10.1038/s41598-025-23676-7

**Published:** 2025-10-28

**Authors:** Krishna S. Nair, Nicholas Hedger, Roana Liz George, Goutam Chandra, Kochupurackal P. Mohanakumar, Bhismadev Chakrabarti, Usha Rajamma

**Affiliations:** 1 Inter University Centre for Biomedical Research & Super Speciality Hospital (IUCBR & SSH), MG University Campus at Thalappady, Rubber Board PO, Kottayam District, Kottayam, 686009 Kerala India; 2 School of Psychology and Clinical Language Sciences, University of Reading, Whiteknights, Reading, RG6 6ES UK; 3 Present Address: Department of Electronics and Biomedical Engineering, Adi Shankara Institute of Engineering and Technology, Ernakulam, 683574 Kerala India

**Keywords:** ADHD, ASD, Eye tracking, Indian population, Social attention, Social behaviour, Social neuroscience

## Abstract

The ability to attend to social stimuli is fundamental for processing social cues and shaping social behavior, yet cultural variability in this capacity remains relatively unexplored. Social attention is typically tested using preferential-looking paradigms in labs, which have demonstrated that autistic individuals attend less to social stimuli. Such studies are limited, by the fact that they have almost all been conducted in Western Europe and the USA. To address this gap, our objective was to test the cultural generalizability of these results by investigating whether autistic symptoms are negatively associated with social attention in a traditionally understudied sample: Indian adults. Additionally, we tested the specificity of this relation by investigating whether a similar association exists with the traits of attention-deficit/hyperactivity disorder (ADHD). Our study involved 121 young adults from Kerala, India. Autistic and ADHD traits were evaluated using the Autism Spectrum Quotient (AQ) and Adult ADHD Self-Report Scale (ASRS), respectively. The participants’ gaze behavior was recorded during a preferential-looking task, where pairs of social and non-social images were presented simultaneously. Individuals with higher autistic traits exhibited a reduced preference for social stimuli. No such association of social attention was noted with ADHD traits. Follow-up analysis of AQ subscales indicated that the association between gaze duration and autistic traits was driven by the social, and not the attention to detail factor of autistic traits. Our results provide new evidence for the cultural generalizability of the social attention task and offer the potential for culture-agnostic phenotypic assessments for adults with autism.

**Supplementary Information:**

The online version contains supplementary material available at 10.1038/s41598-025-23676-7.

## Introduction

Social cognition refers to a set of processes, including perception, interpretation, and response to any socially relevant information, and is influenced by arousal, attention, and emotion, modulating it at various levels^1^. Social cognition provides the bedrock for everyday social interactions, affecting how individuals perceive themselves and others, form relationships, and navigate social situations. Humans display an attentional bias toward social stimuli from an early age, which can have important consequences for social cognitive processes^2^. Preferential orienting to and engagement with social stimuli can provide individuals with more information on social signals from others, which in turn can shape social behavior. Individuals with autism spectrum disorder (ASD) often face challenges in navigating the social world and show atypical visual attention toward social cues in lab-based tasks^3,4^.

Eye tracking is an efficient technique for mapping the explorative patterns of eye saccades and fixations, which provides an online measure of environmental sampling during different tasks^5,6^. This technology offers an effective method for evaluating social cognition and has been extensively used in studies involving individuals with ASD, including children and young adult cohorts^7,8^. These studies focused on understanding how individuals with ASD direct their attention toward social cues, such as faces and social settings, and the impact of joint attention on their social interactions. Notably, these lab-based studies have revealed an association between visual social attention and autism characteristics, demonstrating reduced preferential attention to social cues among individuals with ASD^3,4,8–10^.

One limitation of this literature is that the vast majority of these eye-tracking studies examining visual attention toward social cues in ASD have been observed in Western countries^11–17^. At the same time, it is widely recognized that cultural influences have a significant role in shaping social cognition and behavior^18^, thereby influencing the perception, diagnosis, and support of individuals with ASD^19^. While two relatively recent studies have been conducted on children in Asian countries such as Qatar and China^20,21^, there remains a notable absence of published reports on analogous studies in adults. Research on the intersection of culture and social cognition can provide valuable insights into how cultural factors influence the manifestation of autism-relevant traits^22^. This continued exploration and understanding of cultural influences on autism-relevant phenotypic dimensions can, in principle, lead to more effective and culturally tailored support and diagnostic measures for autistic individuals worldwide.

The present study employs the preferential-looking paradigm, a widely utilized method in developmental psychology, where the participants are presented with competing social and non-social images to assess their attentional bias. This specific paradigm has been tested and validated several times in autistic and non-autistic adults in the UK^12,14,23,24^. Based on these findings, we propose the following hypothesis: (i) the current paradigm will demonstrate cultural generalizability, such that young adults in India will exhibit a positive social attentional bias similar to that observed in samples from Western Europe and the United States, and (ii) higher levels of autistic traits will be associated negatively with attention towards social stimuli.

In addition to testing the cultural generalizability of the results, we explored if the observed effects extend to other dimensional measures of psychopathology, particularly attention deficit hyperactivity disorder (ADHD). Despite potential differences in underlying mechanisms, ADHD is also associated with difficulties in social cognition^25^. Although both ASD and ADHD are associated with social cognitive impairments, the nature and extent of these impairments often differ. For example, differences are observed in domains, including the theory of mind, emotion recognition, and pragmatic language use^26,27^. In this study, we explored the specificity of the relationship between social attention and autistic traits by exploring whether a similar relationship was also seen with traits related to ADHD. This comparative approach allows us to assess the relative influence of autistic and ADHD traits on social attention, thereby discerning whether differences in social attention are specifically linked to autistic traits or if they are indicative of broader attentional patterns related to ADHD. Additionally, this methodology permits an evaluation of the preferential-looking paradigm as a reliable measure of social attention across diverse neurodevelopmental traits.

## Materials and methods

### Subject recruitment

We recruited 121 young adults (mean age: 25.09, SD: 5.43; 52 males) from Kerala, India, and included graduate and postgraduate students or working professionals. All participants had normal or corrected-to-normal vision and no significant medical history, as confirmed by a short clinical interview. Participants were recruited through word-of-mouth referrals and announcements disseminated across various colleges within the university campus. The socio-demographic details of the participants are detailed in Supplementary Table 1. All participants were drawn from within and around the educational institutions and comprised individuals from middle-class and upper-middle-class backgrounds, as assessed by their academic achievements and professional status. Furthermore, all the participants were of Dravidian ethnicity and native Malayalam speakers from Kerala, ensuring cultural homogeneity within the sample.

The sample size for the study was calculated using power analysis based on established literature^3,14^. A medium effect size (*r* = 0.3) was considered appropriate for the analysis. Utilizing a two-tailed correlation test with an alpha level of 0.05 and a desired power of 80%, the required sample size was 82 participants. Given our sample size of 121 participants, the study is adequately powered to detect significant correlations. The study protocol was approved by the institutional ethics committee of IUCBR & SSH (IUCBR-IEC/Certificate/2019-5), and all participants provided informed written consent before their participation. All methods were carried out in accordance with relevant guidelines and regulations. The participants received non-monetary rewards like sweets and stationery items as a gesture of appreciation for their participation.

### Assessment

We evaluated the autistic and ADHD traits of the participants using the Autism Spectrum Quotient (AQ) and the adult ADHD self-report scale (ASRS), respectively. AQ is a 50-item questionnaire that measures an individual’s autistic traits, with higher scores indicating higher autistic traits^28^. Similarly, ASRS is an 18-item tool used to assess ADHD traits in adults, where elevated scores indicate heightened ADHD traits^29^. Both questionnaires have been validated to assess traits in the general population^28,30^. We administered the questionnaire online through a secure web-based link in the English, as all participants were proficient and comfortable with the language. AQ and its corresponding factor scores were computed in accordance with the previous report^31^, while the ASRS scores were calculated as outlined in the original report^29^. Detailed trait score data of the participants are presented in Table 1. To assess the reliability of these measures in the study, we computed Cronbach’s alpha, which indicated acceptable internal consistency for both AQ (=0.69) and ASRS (=0.72). The AQ subscales for social interaction (=0.74) and attention to detail (=0.69) – also showed acceptable internal reliability.

**Table 1:** Descriptive statistics of the demographic (age) and trait score (AQ and its subscale scores and ASRS scores) details. AQ: autism spectrum quotient, ASRS: ADHD self-report scale.

	Minimum and maximum possible score	Range	Mean	Std. Deviation
Age		16–44	25.09	5.431
AQ	50–200	76–141	112.4	11.91
Social interaction factor of AQ	40–160	56–112	87.09	11.33
Attention to detail factor of AQ	10–40	13–40	25.34	5.272
ASRS	0–18	0–17	5.289	3.282

Among the 121 participants, two individuals were clinically diagnosed with ADHD. No participant had a documented history of intellectual disability or cognitive impairment, are verified through self-report data and a brief clinical interview conducted by trained professionals.

### Stimuli

Each trial in the preferential-looking task involved the simultaneous presentation of two images - one social and one non-social - displayed side by side on the screen. The study incorporated a total of 30 trials, where each pair of images was presented using MATLAB with Psychtoolbox extensions^14^. Social images included depictions of humans, such as images of infants, couples, etc., while non-social images featured items such as food, natural scenery, and various objects. We used the same image set as in Hedger & Chakrabarti et al., 2021, with images drawn from the International Affective Picture System^32^ and Flickr^33^. To ensure that the image pairs were matched as closely as possible on dimensions other than their sociality, a validation study by Chakrabarti et al. (2017)^11^ reported the affective (valence, arousal) and salience (RMS contrast, luminance, Koch Salience) parameters of all the image pairs.

Also, to minimize the influence of low-level visual elements such as contrast and color on the participants’ visual preferences during the experiment, a phase-scrambled version of each of these image pairs was also included as additional trials in the task^23^. This resulted in a total of 60 trials, 30 intact and 30 scrambled images presented in the same pseudorandom sequence. The spatial positions of social and non-social images were counterbalanced across the experiment.

### Data collection

**Fig. 1:**
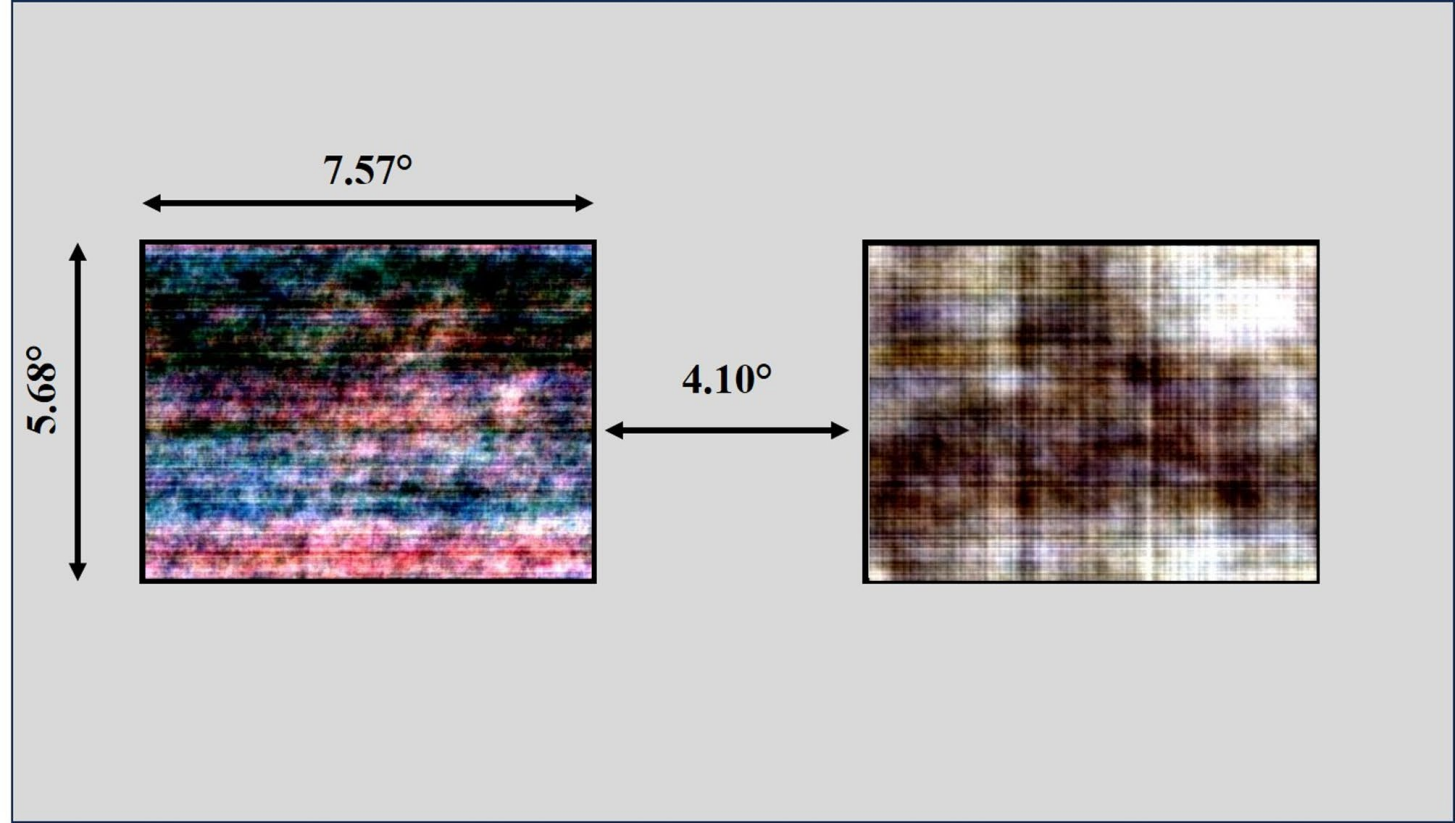
Layout of the trial presentation setup. The display had a resolution of 3.33 pixels/mm. A pair of scrambled images is given here.

**Fig. 2:**
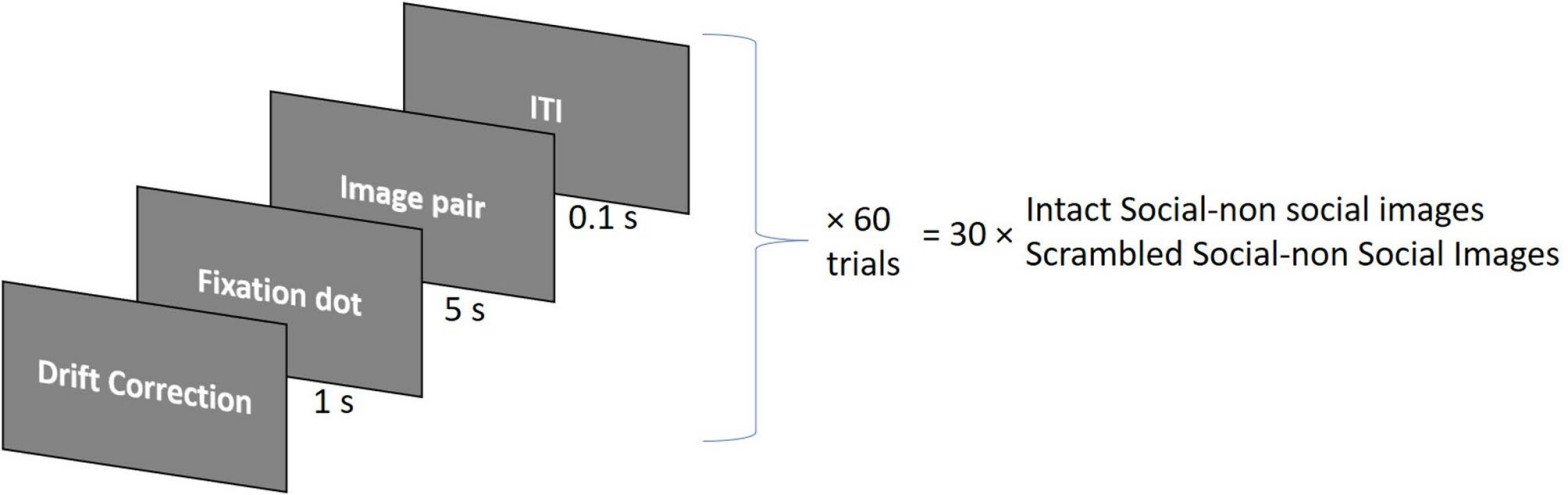
Schematic diagram of the trial sequence. Each trial started with a drift correction, followed by the display of a central fixation dot. Subsequently, a pair of social/non-social images was presented. The next trial started after an inter trial interval (ITI). Thiry pairs of intact images and their corresponding scrambled images were displayed in a predefined pseudorandom sequence.

During the experimental procedure, participants were seated at a distance ranging from 720 mm to 820 mm from the screen. Eye movements and fixations were recorded binocularly at 1000 Hz using an Eyelink 1000 plus eye tracker from SR Research Ltd. The stimuli were presented on a 410 × 230 mm screen with a resolution of 1366 × 768-pixels and a refresh rate of 60 Hz, as illustrated in Fig. 1. Prior to the start of data collection, five-point calibration and validation processes were performed, and the process was repeated if there was a general difference of more than one degree of visual angle between the calibration and validation. Each trial began with a drift correction to confirm that the calibration was still valid. Then, a central fixation dot was presented for 1000 milliseconds (ms), followed by the social/non-social image pairs for 5000 ms. The next trial followed after an inter-trial interval (ITI) of 100 ms. A schematic illustration of the trial sequence is shown in Fig. 2. The participants were briefed that they would be presented with some images and were instructed to relax and take a good look at the presented images. The entire data collection process took 10–15 min.

### Analysis

The eye-tracking data were extracted using the Data Viewer software of SR Research Ltd. The display coordinates occupied by the social and non-social images during stimuli presentation were utilized to define their respective areas of interest (AOI). The Area of Interest (AOI) was defined as the entire region occupied by each image, with each trial encompassing two AOIs, ensuring that comprehensive gaze measurements, capturing all fixations within the designated boundaries of the presented stimuli. Dwell time, defined as the duration in milliseconds participants spent looking at each AOI, was calculated for every trial and subsequently averaged across participants. Trials with over 60% track loss by participants were excluded from the analysis. Consequently, 224 of the 7260 trials (3.08%) were discarded, equating to an average of 1.87 trials removed per participant.

A linear mixed model analysis was implemented using Jamovi^34^ to examine the impact of predictors on dwell time (DT). The model included age, sex (male, female), AQ score, ASRS score, stimulus type (intact, scrambled), and AOI (social, non-social) as fixed effects and random intercepts per subject (ID). The restricted maximum likelihood (REML) method was utilized to estimate the model fit.

The model equation was as follows:

DT ~ 1 + Age + Sex + Stimulus + AOI + AQ + ASRS + Stimulus: AOI + Stimulus: AOI: AQ + Stimulus: AOI: ASRS + (1 | ID).

To ensure the validity of the assumptions underlying the linear mixed model, we assessed the normality of residuals using the Kolmogorov-Smirnov test. The results demonstrated no significant deviation from normality (D = 0.04, *p* = 0.486). In the original analysis, the covariates, age, AQ, and ASRS scores were not scaled. To improve the interpretability of the results, we subsequently reanalyzed the data after scaling the covariates to the mean. The findings from the reanalysis were consistent with those from the original analysis, thereby confirming that mean-centering the covariates did not influence the primary outcomes.

We conducted a follow-up correlation analysis to explore whether the observed effect of autistic traits on dwell time was driven by the social or non-social factors of the AQ. The correlation matrix for the dwell time on the social AOI and AQ factor scores was obtained using GraphPad Prism^35^.

Further, to examine whether the impact of the AQ score on dwell time was influenced by the low-level image characteristics of the presented stimuli, we carried out a manipulation assessment. Specifically, we calculated the structural similarity index measurement (SSIM) for the social/non-social image pairs using MATLAB. The SSIM metric evaluates the likeness between two images by considering essential low-level features like luminance, contrast, and structural information^36^. This methodological choice ensures that any observed differences in dwell time are attributable to the images’ social content rather than inherent differences in their low-level visual properties between the specific social and non-social images within each pair. In our manipulation check analysis, we employed the following model:

Intact Social DT ~ 1 + SSIM + ‘Trial Number’ + AQ + SSIM: AQ+ ‘Trial Number’:AQ+ (1 | ID).

## Results

**Fig. 3:**
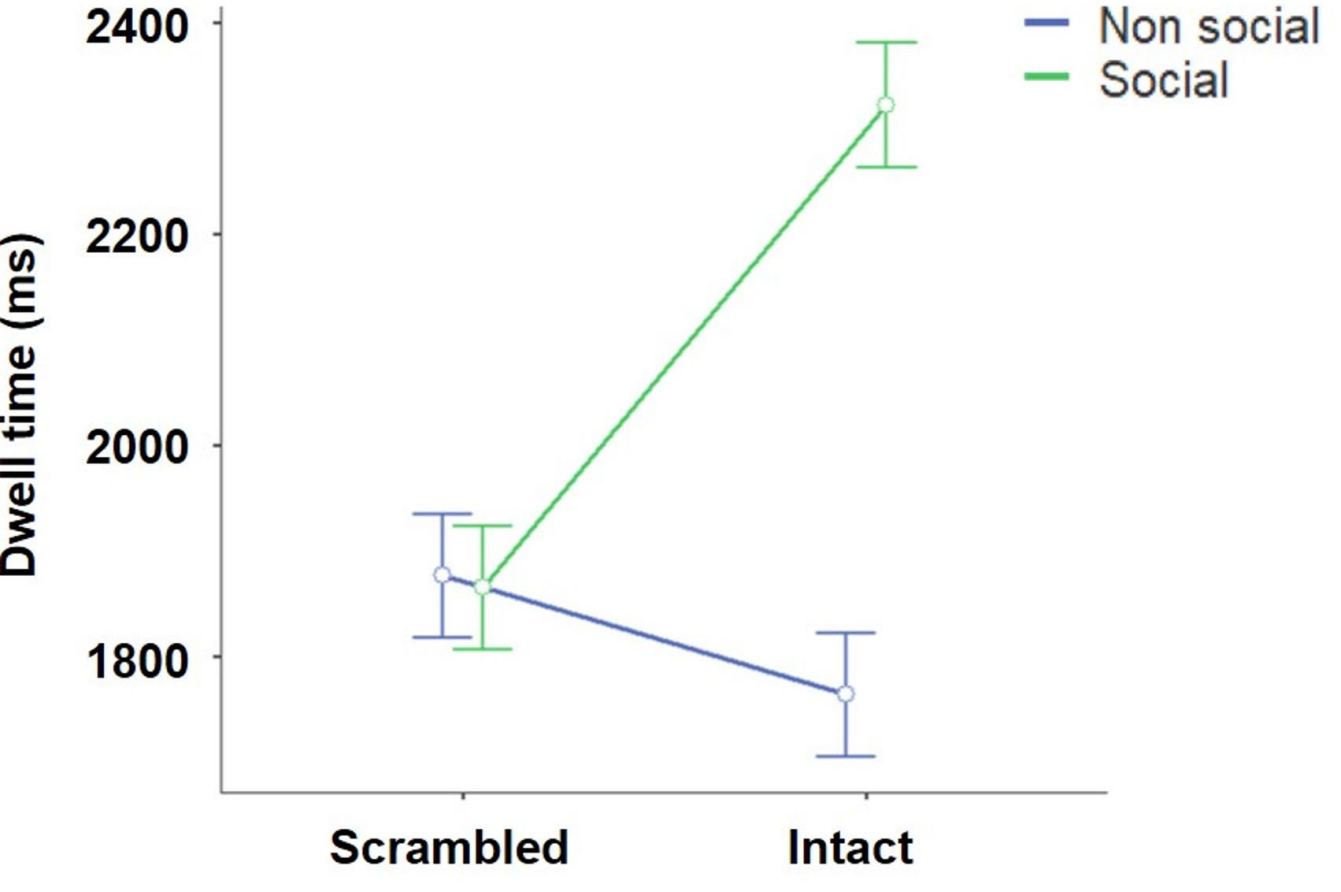
The effect of stimuli and AOI on dwell time. While scrambled images did not affect the dwell time (in ms), the intact images significantly affected the dwell time while viewing social and non-social images with a strong bias towards social image. The error bar signifies 95% confidence interval.

**Fig. 4:**
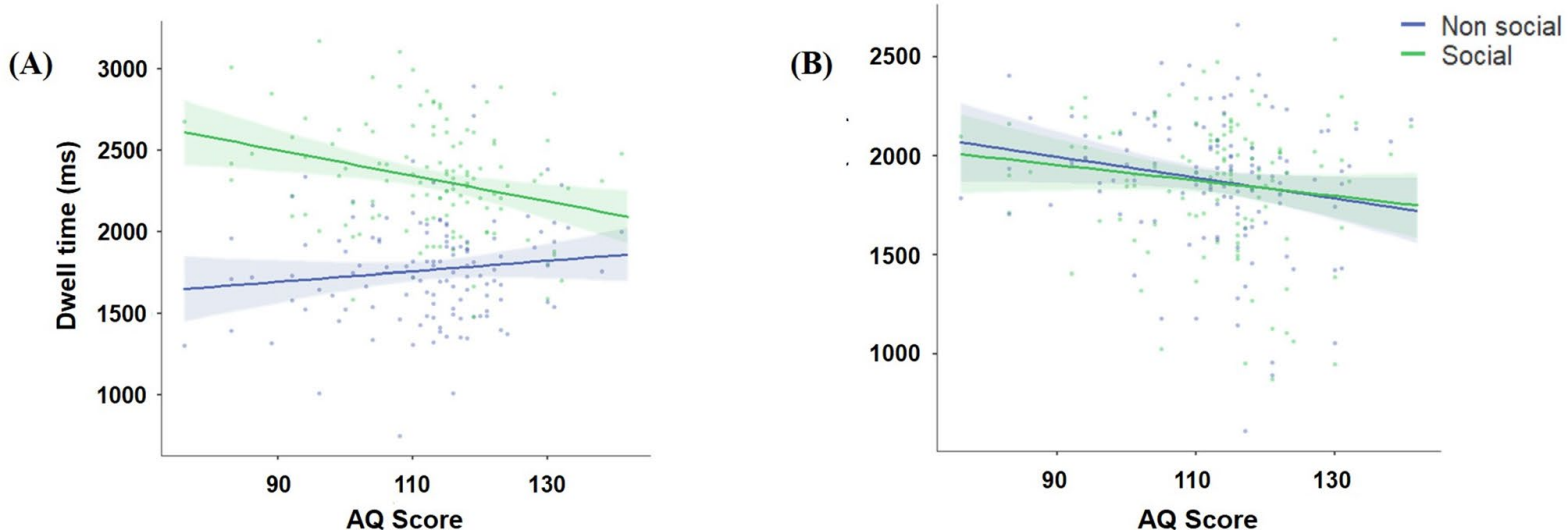
Effect of AQ score on the dwell time in social and non-social area of interest (AOI) for intact and scrambled images. The effect of AQ score on the social and nonsocial AOI (**A**) for intact images displayed an opposite correlation while viewing the two AOIs, (**B**) for scrambled images, the effect of AQ score on the dwell time of social and nonsocial images displayed a similar effect. The shaded error bar signifies 95% confidence interval and the dots represents individual data points.

The outcome of the linear mixed model analysis is presented in Table 2. The analysis indicates a statistically significant main effect of AOI and AQ scores, while no significant effects were observed for age, sex, stimulus type, or ASRS score on dwell time. The analysis revealed a significant interaction effect between stimulus type and AOI. Post hoc comparisons performed between AOI and stimulus type demonstrated that there was a significant difference in dwell time between intact social and non-social images [*t(354) = −14.28*,*p ≤ 0.001*] with a strong bias towards the social image. Conversely, no significant difference was evident between scrambled social and non-social stimuli [*t(354) = 0.29*,*p = 1.000*], as depicted in Fig. 3. Moreover, a significant three-way interaction between the stimulus type, AOI, and AQ was identified. No effect of age or sex was noted in this analysis. Specifically for intact stimuli, the AQ score showed a negative correlation with the social dwell time and a positive correlation with non-social dwell time, as illustrated in Fig. 4A. However, no such differences were observed with scrambled stimuli, as shown in Fig. 4B. The analysis did not reveal a significant interaction effect between stimulus type, AOI, and ASRS.

**Table 2:** Parameter estimates for the fixed effect omnibus tests.

Fixed Effects Omnibus Tests				
	F	df	df (res)	*p*
Age	0.8427	1	116	0.361
Sex	0.3318	1	116	0.566
Stimulus	0.0398	1	354	0.842
AOI	9.7659	1	354	0.002
AQ	6.6152	1	437	0.01
ASRS	0.0933	1	434	0.76
Stimulus ✻ AOI	13.958	1	354	< 0.001
Stimulus ✻ AOI ✻ AQ	3.9169	3	354	0.009
Stimulus ✻ AOI ✻ ASRS	0.3661	3	354	0.778

**Fig. 5:**
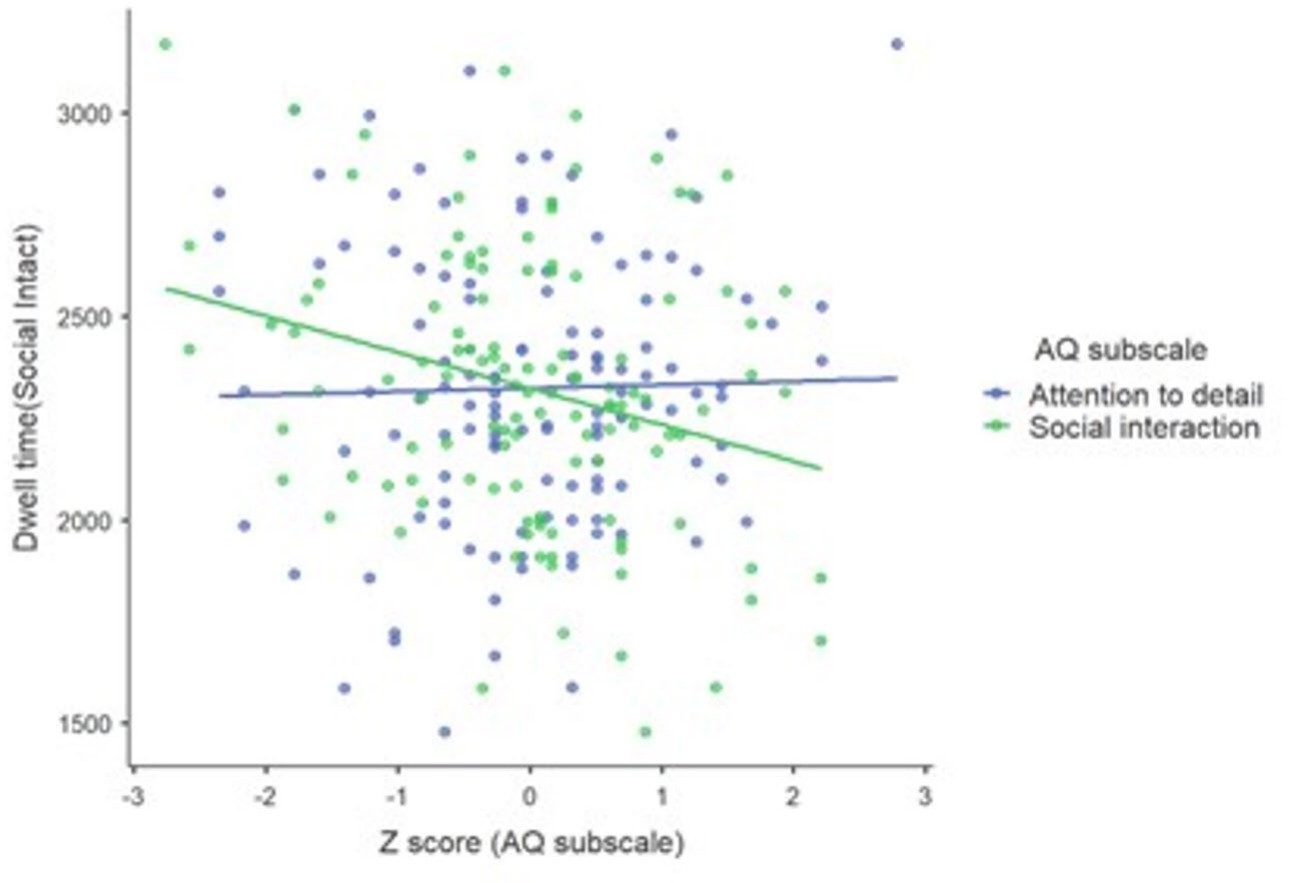
Scatter plot illustrating the relationship between AQ sub-factors and social dwell time.

To test whether the significant association of dwell time with AQ was driven by one or both of the sub-factors of the AQ (social interaction and attention to detail), exploratory correlation analyses were conducted. The results revealed that the inverse relationship between the AQ scores and social dwell time was predominantly influenced by the social interaction factor (*r=−0.26*,*p = 0.004*) rather than the attention to detail factor [*r = 0.02*,*p = 0.789*]. Notably, the disparity between these two correlations was statistically significant [*Steigers Z= −2.06*,*p = 0.039*]. Figure 5 illustrates the distinct relationship between social dwell time and the two AQ sub-factors, emphasizing their differential association with social attention.

The manipulation check analysis to investigate the influence of low-level image properties on dwell time in social AOI revealed that neither the SSIM scores of the image pair [*F(1*,*3480) = 0.17*,*p = 0.682*] nor the interaction effect between AQ scores and SSIM scores [*F(1*,*3480) = 0.02*,*p = 0.880*] had a significant effect on dwell time on the social images. Interestingly, the trial number had a significant positive effect on dwell time on the social image [*F(1*,* 3480) = 24.42*,*p < 0.001*]. In a previous study, we observed that preferential social attention declines over trial time^14^, as a function of autistic traits. To test whether autistic traits similarly influenced preferential social attention through the course of the experiment, the interaction effect between AQ score and trial number was examined. The analysis revealed that the interaction did not produce a significant effect *[F(1*,* 35) = 0.65*,*p = 0.421*]. The findings indicate that the relationship between AQ score and dwell time was stable across the trials, suggesting that the influence of AQ on social attention remained unchanged throughout the experiment.

## Discussion

The present study aimed to investigate the relationship between autistic and ADHD traits and social visual attention among young adults in India using a preferential-looking paradigm. Specifically, we assessed the association between autistic traits, measured by the AQ score, and ADHD traits, as assessed by the ASRS, in relation to attentional bias toward social stimuli. Our findings revealed a significant negative association between autistic traits and social attentional bias, while ADHD traits did not demonstrate a significant relationship with social attention. These results not only replicate previous research conducted in the UK using the same experimental paradigm^14^ but also underscore the cultural generalizability of the preferential-looking paradigm as an effective tool for assessing social attention across diverse population.

Across the whole sample, participants demonstrated a strong bias towards social images, as reported in previous studies with this paradigm in UK samples^14^. To test if this observed social bias was driven by low-level stimulus properties, two checks were implemented. First, phase-scrambled versions of the same image pairs were presented to the participants. No attentional bias was noted for the social images in phase scrambled versions. Second, structural similarity index measurements were calculated for each intact image pair. This metric showed no relationship with the observed attentional bias for social images. Together, there is sufficient strength of evidence to suggest that social attentional bias in this sample cannot be explained by stimulus properties alone.

The observed negative relationship between autistic symptoms and social attentional bias replicates findings from several studies that have used similar preferential-looking paradigms in autistic and nonautistic samples primarily in Western Europe and USA^3,4^. While the majority of these studies have been conducted in children, a similar pattern has also been observed in autistic and nonautistic adults^14^. Recent efforts to study similar paradigms in Asian samples involve using culturally appropriate stimuli^20,21^. The current study presents three points of contrast from these recent efforts. First, it uses the same stimuli as used in the UK sample, thus demonstrating that the results are generalizable in an Indian sample even without cultural adaptation of the stimuli. This generalizability could, in principle, be attributed to the relatively high familiarity of the white faces and images via online and social media platforms and television. Second, both of these recent Asian studies were conducted in children. The current study shows that the results are generalizable to an older young adult sample. Third, neither of these previous studies have tested the potential impact of low-level stimulus features in the observed results.

Further investigation of the negative relationship between autistic traits and social attention revealed that this effect was primarily driven by the social dimension of the autistic traits, rather than the attentional factor. This finding is consistent with studies in children that suggest that reduced attention on social stimuli may be indicative of difficulties in social communication and interaction^37^. Social cognition differences, as measured by visual preference and joint attention, have been shown to be associated with social affect scores of ADOS, rather than scores related to restricted and repetitive behaviors^21,38^. This supports the notion that autistic traits encompass multidimensional constructs that do not uniformly impact social cognition^39^. Given the potential variability in factor structures across different translations and demographical groups, future research should focus on validating the sub-factors of the AQ in diverse populations to confirm the observed associations with social attention.

Our findings are consistent with previous neurodevelopmental research that identifies atypical social attention as a prevalent feature of ASD. The underlying origins of these differences in social attention remain speculative. While common genetic variations could theoretically contribute to modest initial differences in social attentional patterns, the ultimate manifestation of this phenotypic marker is likely modulated by developmental processes. For instance, individuals with a reduced initial preference for social stimuli may engage less with social signals, subsequently leading to reduced learning from these interactions. This impairment in social learning may precipitate heightened anxiety in social situations, prompting individuals on the spectrum to actively avoid social stimuli in the future. Measuring neural response to social rewards in adulthood has indicated alterations within the social neural circuitry in autism^40^. However, similar to the differences in social attention observed in our study, these neural differences, like the differences in social attention in the current study, likely reflect the product of genetic predisposition and developmental experience. Remarkably, the lack of a significant correlation between social dwell time and attention to detail subscores suggests that the differences in social attention are more directly related to the variations in social interest and processing abilities, rather than indicative of sensory differences^14,41^.

Interestingly, no relationship was noted between social attentional bias and ADHD symptoms. While both autism and ADHD are often associated with difficulties in social cognition^25,42^, their underlying symptoms may manifest differently in lab-based tests of social attention. These findings also support previous research indicating that alterations in social visual attention measures are specific to ASD and are not evident in ADHD^43^.

In the current study, no significant effects of age and sex on social attentional bias was noted, which is consistent with the largest meta-analysis on social orienting paradigms that reported a null effect of age and sex^4^. While age and sex were not the primary variables of interest in this study, they were included in the model to account for potential individual differences in social attention. The absence of significant effects further reinforces the specificity of the observed relationship between autistic traits and social attention, suggesting that the identified effects are unlikely to be confounded by broader demographic variables.

Both assessment scales used in our study are internationally recognized evaluation tools. Our findings corroborate with the existing studies, confirming the reliability of AQ and ASRS are well-established evaluation instruments within the Indian population. Specifically, the AQ had good internal consistency, as reported by Karmarkar et al. (2021)^44^, who examined the distribution of autistic traits among Indian adults. Similarly, ASRS exhibited commendable internal consistency, thereby affirming its validity as a measure for ADHD characteristics in this group.

While demonstrating the feasibility and generalizability of the employed paradigm in an Indian sample, the study presents several opportunities for building on these preliminary findings. First, dynamic social stimuli, particularly those involving interactive elements are known to elicit more pronounced effects in social attentional bias^4,21^. Therefore, future studies should move beyond relying exclusively on static stimuli to index social attentional bias. Second, given the use of convenience sampling, our participant group likely represents higher-functioning individuals with ASD traits, which may limit the generalizability of the findings to the broader ASD population. Third, it is important to note that the current sample primarily consisted of a non-autistic population, except for the two clinically diagnosed cases. Subsequent studies should aim to test if these findings can be generalized to autistic populations, particularly focusing on the understudied subgroup of nonverbal or minimally verbal individuals within a culturally underrepresented population. Lastly, although participants in this study did not report a prior clinical diagnosis of anxiety or express anxiety-related concerns, we did not measure anxiety levels due to time limitations. Given the established influence of anxiety on social behaviors and eye contact^45^, future research should incorporate measures of anxiety. By addressing these areas, subsequent studies can enhance our understanding of social attentional processes and their potential effects on eye contact and social attention.

## Conclusion

The current study demonstrates the generalizability of a preferential-looking paradigm to index social attentional bias in an Indian young adult sample. Our findings indicate a significant negative correlation between autistic traits and social attentional bias. In contrast, no such relationship was observed for traits associated with ADHD. These results highlight the potential for creating culture-agnostic, performance-based assessments that effectively measure the autistic phenotype utilizing scalable methodologies^46^.

## Supplementary Information

Below is the link to the electronic supplementary material.

Supplementary Material 1

## Data Availability

Data that support the findings of this work are available from the corresponding/co-corresponding author, upon reasonable request.
