# Supplementary material for: Eye tracking demonstrates the influence of autistic traits on social attention in a community sample from India
Source: Sci Rep. 2025 Oct 28;15:37552. doi: 10.1038/s41598-025-23676-7 (PMC12569073; doi:10.1038/s41598-025-23676-7)
Supplement: Supplementary file 1 — Supplementary Material 1 [file 41598_2025_23676_MOESM1_ESM.docx]

Supplementary Table 1: Socio-demographic profile of the recruited participants.

| **Characteristic** | **Item** | **Number of participants** | **Percentage of participants** |
| --- | --- | --- | --- |
| **Gender** | Male | 52 | 42.98 |
|  | Female | 69 | 57.02 |
| **Age** | 16-22 | 43 | 35.54 |
|  | 23-29 | 56 | 46.28 |
|  | 30-36 | 18 | 14.88 |
|  | 37-44 | 4 | 3.30 |
| **Employment** | Employed | 43 | 35.54 |
|  | In Education | 78 | 64.46 |
| **Employed: Highest qualification** | Undergraduate | 28 | 65.12 |
|  | Post Graduate | 9 | 20.93 |
|  | Doctorate | 6 | 13.95 |
| **In education: Pursuing degree** | High School | 3 | 3.84 |
|  | Undergraduate | 37 | 47.44 |
|  | Post Graduate | 23 | 29.49 |
|  | Doctorate | 15 | 19.23 |
| **Handedness**  **(as measured by Edinburgh Handedness Inventory)** | Right | 120 | 99.17 |
|  | Left | 1 | 0.83 |
